# Sulfur isotope engineering in heterostructures of transition metal dichalcogenides†

**DOI:** 10.1039/d4na00897a

**Published:** 2025-01-13

**Authors:** Vaibhav Varade, Golam Haider, Martin Kalbac, Jana Vejpravova

**Affiliations:** a Department of Condensed Matter Physics, Faculty of Mathematics and Physics, Charles University Ke Karlovu 5, 12116, Prague 2 Czech Republic jana.vejpravova@matfyz.cuni.cz; b Department of Low-Dimensional Systems, J. Heyrovsky Institute of Physical Chemistry Dolejskova 3, 18223, Prague 8 Czech Republic martin.kalbac@jh-inst.cas.cz

## Abstract

Heterostructuring of two-dimensional materials offers a robust platform to precisely tune optoelectronic properties through interlayer interactions. Here we achieved a strong interlayer coupling in a double-layered heterostructure of sulfur isotope-modified adjacent MoS_2_ monolayers *via* two-step chemical vapor deposition growth. The strong interlayer coupling in the MoS_2_(^34^S)/MoS_2_(^32^S) was affirmed by low-frequency shear and breathing modes in the Raman spectra. The photoluminescence emission spectra showed that isotope-induced changes in the electronic structure and strong interlayer coupling led to the suppression of intralayer excitons, resulting in dominant emission from the MoS_2_(^32^S) layer. Time-resolved photoluminescence experiments indicated faster lifetimes in the MoS_2_(^34^S)/MoS_2_(^32^S) heterostructure compared to the conventional bilayers with the natural isotopic abundance, highlighting nuanced interlayer exciton dynamics due to the isotopic modification. This study underscores the great potential of isotope engineering in van der Waals heterostructures, as it enables tailoring the band structure and exciton dynamics at the nuclear level without the need of chemical modification.

## Abstract 2

Isotope-engineered MoS_2_ bilayers, composed of ^34^S and ^32^S provided access to the top and bottom layer individually. Raman and PL studies highlight suppressed intralayer excitons and nuanced exciton dynamics due to the isotopic modification.

## Graphical abstract

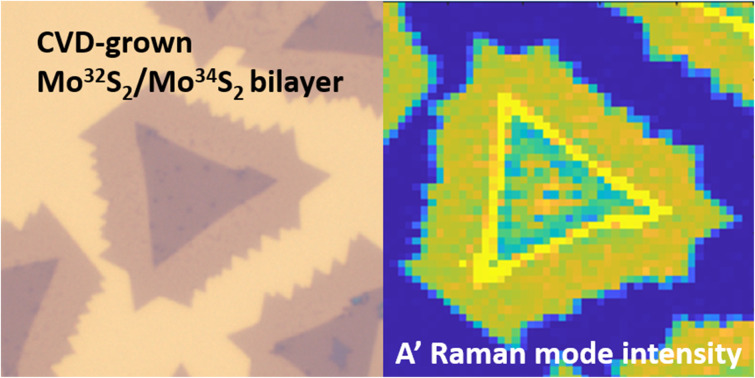

## Introduction

Since the discovery of graphene, there has been considerable growth in the genre of two-dimensional (2D) materials.^1,2^ In this direction, the family of 2D transition metal dichalcogenides (TMDCs) has brought much excitement as well as puzzles due to their unique physical properties, offering a huge scope in manipulating their response thanks to the multimodal coupling between the optical, electrical, and mechanical properties.^3–5^ 2D TMCDs exhibit strong light-matter interactions giving rise to the enhanced photoluminescence (PL), non-trivial exciton formation, and nonlinear optical effects.^6–8^ This makes them highly attractive for integration into optoelectronic devices, including photodetectors and light-emitting diodes.^9,10^

The unique properties of TMDCs can be further advanced by their smart assembly into artificial van der Waals (vdW) crystals, *i.e.*, by creating vertical or lateral heterostructures (HS).^11^ In the vertically assembled HS, the interaction between the layers gives rise to novel phenomena such as interlayer excitons, where an electron and a hole are located in adjacent layers.^12^ By controlling the interlayer coupling, one can tailor the band alignment, excitonic properties, and charge transfer dynamics, which is essential for the precise control over the device functionalities in various optoelectronic and valleytronics applications.^13,14^ In addition, a small twist angle or lattice mismatch between the layers give rise to the moiré superlattices with a periodic variation in the interlayer alignment leading to strong correlations and exciton localization.^14–16^ All these unique features, in addition to the valley-driven physics^17,18^ make the TMCD-based vertical HS versatile components of the next-generation electronic, optoelectronic, and quantum devices.^19–21^

The stacking order in TMDCs bilayers significantly influences their electronic properties and band structure. In particular, the interlayer interactions, which are dependent on the stacking configuration, modify the electronic coupling between layers, leading to different charge density distributions and enable to switch between the direct and indirect band gap, *etc.* There are some specific cases of the stacking:

AA-stacking (or direct stacking): in this configuration, the layers are perfectly aligned, with the atoms in one layer directly above the atoms in the other. This configuration typically results in weak interlayer coupling and can lead to a direct bandgap. The electronic structure tends to resemble that of a monolayer in terms of the bandgap and excitonic properties.

AB-stacking (or rhombohedral stacking): in this arrangement, the two layers are shifted relative to each other, creating a structure where atoms in the top layer are above the chalcogen atoms in the bottom layer. This configuration typically results in stronger interlayer interactions, leading to the bulk-like indirect bandgap. The interlayer coupling is stronger here than in the AA-stacked configuration, which can also influence exciton dynamics.

AB'-stacking (or twisted stacking): this configuration occurs when the layers are rotated relative to each other, introducing a twist angle between the layers. This introduces a periodic moiré pattern and can significantly modulate the electronic properties. At certain twist angles, this stacking can lead to enhanced electron–electron interactions and potentially induce phenomena such as the emergence of flat bands or correlated insulator phases, similar to what is observed in twisted bilayer graphene.

In these special stacking cases, the interlayer interactions lead to diverse electronic behaviors that can be fine-tuned for specific applications, such as in optoelectronics, transistors, or quantum computing. The manipulation of stacking orders thus becomes an important tool for designing materials with tailored properties.

In this context, it is crucial to understand the impact of the charge, strain and light driven electronic coupling in the vertical HS, as the interlayer interactions profoundly influence the coveted opto-electronic properties of the HS.^22^ Therefore, addressing individual layers in the HS is essential. This is in general possible only in case that the constituent TMDCs are composed of the different chemical elements, which always brings additional complexity to the system. Thus, to study the pristine nature of the interlayer coupling, a smart tool enabling selective addressing of the individual layers even without changing the chemical composition is of high demand.

In this vein, isotope engineering provides a unique platform to modify the physical properties of without altering the chemical composition which has been demonstrated successfully in various systems.^23,24^ In 2D materials, such as graphene and TMDCs MLs, it has been also observed that the thermal, electronic, and vibrational properties are significantly modified by introducing isotopes of the respective elements, such as carbon and tungsten.^25^ Recently, it has been shown that the lattice and excitonic properties of MoS_2_ MLs can be effectively tuned by varying the type and ratio of the sulfur isotopes.^26^ As TMDCs are extensively studied 2D materials, introducing the concept of chalcogen isotope engineering can precisely illustrate how the phonon-driven properties can be tuned down to the subatomic level. Therefore, utilizing the isotope engineering in vertical TMDC-based HS can significantly expand the possibilities of fine-tuning their properties with the possibility of addressing the individual layers.

Until now, there have been only a few studies on isotope modification in layered HS.^26–28^ For example, in laterally structured HS of isotope-modified MLs, an unusual red shift in the optical bandgap energy with an increase in the mass of Mo isotopes was observed.^28^

From the perspective of the HS fabrication, there is a persisting challenge to achieve a close-to-ideal interface between the layers. Despite significant advancement of the transfer methods in recent years,^29^ the heterostructuring of the mechanically exfoliated monolayers (MLs) often results in deteriorated coupling between them due to the insufficient quality of the interface. It has been demonstrated that vertical HS can be grown using the controlled molecular beam epitaxy (MBE) method.^30^ While the quality of the interface appears to be sufficient, the MBE technique is not a very cost-effective for routine production of high-quality HS. A convenient alternative with a very good cost-to-quality ratio is the stepwise chemical vapor deposition (CVD) technique. It has been shown that this method can provide vertically stacked, strongly interacting layered HS even on the wafer scale.^31^

In this work, we succeeded in the growth of the vertical HS of sulfur isotope-modified MoS_2_*via* a two-step CVD method. This enabled us to create bilayer structures without altering the chemical composition of the individual layers. Nevertheless, introducing the different sulphur isotopes enabled us to address both layers selectively by extensive Raman and PL spectroscopy studies. A thorough analysis of the Raman spectral maps revealed a clear shift of the Raman active modes towards lower wavenumbers with increasing mass of the sulfur isotopes and unexpectedly strong coupling between the layers. We also observed that the emission pathways in PL of isotope-modified HS can be altered due to the variation of the band gap in the adjacent layers differing in the sulfur isotope mass. Our study unambiguously highlights the significance of the isotope engineering as a powerful tool for tuning the physical properties of 2D materials on the subatomic level, opening new avenues for advanced materials design and applications.

## Experimental

The double-layered HS of two different isotopically-labeled MoS_2_ MLs (with ^32^S and ^34^S) were fabricated *via* two-step CVD growth on Si/SiO_2_ substrate. For the first ML, MoO_2_ (60 mg) was placed in a quartz crucible along with SiO_2_/Si substrate. The substrates were thoroughly cleaned *via* sonication in deionized water, acetone, and isopropanol (Sigma-Aldrich). The crucible was then inserted in the middle of a 40 cm long quartz tube of 15 mm in diameter. Thereafter, 100 mg of pure ^32^S was placed in the tube 20 cm apart from the crucible and the tube was kept in a bigger quartz tube of length 80 cm and diameter 25 mm. The tube was connected to an argon gas line on one end and to a bubbler filled with 100 mM aqueous solution of KOH. The tube was flushed with argon for 15 minutes, and afterward, part of the tube containing the crucible was heated under a constant flow of argon (120 cm^3^ min^−1^) in a cylindrical furnace at the rate of 40 °C min^−1^. When the temperature reached 770 °C, the sulfur was introduced into the furnace by shifting the tube. After reaching 820 °C, the temperature was kept constant for 10 minutes, after which the furnace was opened, and the system was left to cool down. After obtaining the substrate was immediately placed in another cleaned crucible and the same process was repeated for ^34^S with fresh quartz tubes to avoid any contamination. In addition, pristine MLs and BLs of both sulfur isotopes were synthesized separately in the one-step CVD method for obtaining a comparison.

AFM images and thickness profiles were obtained using Bruker's AFM Dimension ICON system in the quantitative non-mechanical mode with a Bruker silicon tip. The AFM data were processed and analyzed by Gwyddion software.^32^

Optical and PL images (under green light illumination) were obtained using an Olympus microscope. The ambient Raman/PL spectral maps were recorded using a WITec Alpha300R spectrometer equipped with a piezo-stage and a RayShield Coupler with a laser power of 100 μW (532 nm) for all the samples. All the spectral maps (20 × 20 μm^2^) were scanned with 0.5 μm/line resolution using a 100× objective.

The reproducibility and reliability are ensured through the mapping approach and repeating the experiment at different positions on the sample. In a scanned area of 20 × 20 μm^2^, with a spatial resolution of 0.5 μm in one dimension, we record 400 spectra. Even if the scanned area contains two distinct regions (*e.g.*, monolayer and bilayer), we still obtain about 200 spectra for each region. Since monolayers and bilayers are layered crystals, compositional and structural variations below the laser spot size are negligible. The variation in Raman shifts and exciton positions is within the instrumental resolution, approximately 1 cm⁻^1^ absolutely and for the Raman spectra, and in order of meV for the PL, respectively.

The time-resolved (TR) PL mapping of the individual MoS_2_(^32^S) and MoS_2_(^34^S) MLs and the MoS_2_(^32^S)/MoS_2_(^34^S) HS were performed on an Olympus FluoView1000 confocal microscope integrated with a PMT detector (tau-SPAD, PicoQuant) of sub-nanosecond TCSPC capability (HydraHarp 400, PicoQuant) with 2.33 eV pulsed laser excitation at a laser power of 25 μW, frequency 1 MHz, and pulse duration of 50 ps. For the spatially resolved measurement, the photon arrival times of each recorded photon for the previous laser pulse were stored using ultra-fast electronics in a time-tagged TR recording mode in a fluorescence lifetime imaging microscopy images approach, in which every given pixel resembles the average photon arrival time. Finally, the TR emission spectra and excited state carrier lifetime were obtained using an iterative deconvolution process with PicoQuant Fluorite software convoluted with the experimental instrument response function (IRF) ∼125 ps.

The PL and Raman spectral maps were analyzed using Project FIVE, ORIGIN, and a homemade routine in Matlab software. The individual Raman and PL modes were fitted with pseudo-Voigt functions corresponding to respective phonon modes and excitons. The Lorentzian component and the Gaussian component are used to account for the intrinsic profile and distribution of the peak(s) parameters of the specific area, respectively. The resulting peak parameters were used in the Raman correlation analysis.

## Results and discussion

**Fig. 1:**
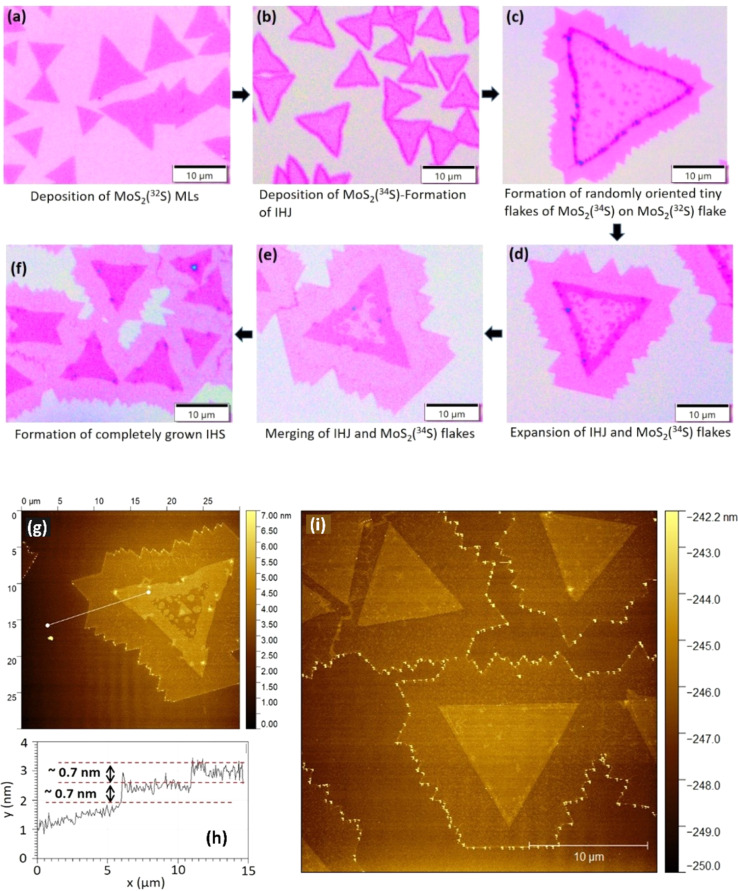
Optical and AFM images of different structures grown *via* two-step CVD. (a–f) Shows the crucial phases of the growth, starting from the MoS_2_(^32^S) ML (a), propagating to the IHJ (b) and finally the HIS (d–f), respectively. (g) AFM image of partially grown HS along with its (h) thickness profile along the white marked line. (i) AFM topographical image of a completely grown layered IHS of MoS_2_(^34^S)/MoS_2_(^32^S).

The layered HS were successfully fabricated *via* a two-step CVD process on the SiO_2_/Si substrates. The obtained structures are shown in Fig. 1(a–f). Initially, a MoS_2_ ML with ^32^S isotopes was synthesized, resulting in well-defined triangular crystals (Fig. 1(a)). In the subsequent CVD step the MoS_2_ MLs with ^34^S isotopes were grown. The second layer is typically nucleated at the edges of the first layer of the MoS_2_(^32^S) triangles, forming a thin heterojunction, as indicated in Fig. 1(b). The dark purple regions correspond to the formation of isotopic heterojunctions (IHJ) of a typical width of less than 1 μm, which are resulting from a vertical overlapping of the MoS_2_(^32^S) and MoS_2_(^34^S) layers. Simultaneously, tiny MoS_2_(^34^S) triangles with a random orientation are formed on top of the MoS_2_(^32^S) triangles (Fig. 1(c)). As the growth progresses, the IHJ expanded both inward and outward, with the outward growth producing pure MoS_2_(^34^S) regions, and inward growth leading to the expansion of the MoS_2_(^34^S) layer over the MoS_2_(^32^S) base (Fig. 1(d and e)). Furthermore, the tiny MoS_2_(^34^S) triangles also grow in size and eventually merge with the expanding IHJs, covering the initial MoS_2_(^32^S) triangles entirely, forming isotopic HS (IHS) as shown in Fig. 1(f). The final size of the different MoS_2_ crystals varies in the range between 10 and 30 μm.

Fig. S1† shows PL maps matching the optical images of the pristine MLs, IHJ, and IHS regions, respectively. The growth process resulted in complex HS with various stacking arrangements, including AA, AB, and other twist angle configurations (discussed further). The stacking of the layers was carefully monitored as the twist angle in bilayers (BLs) plays an important role in manipulating the intrinsic properties of the structures.^33,34^

The samples of IHS were further imaged by AFM, as shown in Fig. 1(g–i). Fig. 1(h) shows the thickness profile across the white line shown in the Fig. 1(g), which confirms that the IHS is composed of MoS_2_ MLs (thickness of each ML is about 0.7 nm). Finally, a completely grown IHS can be seen in the topographic AFM image shown in Fig. 1(i).

**Fig. 2:**
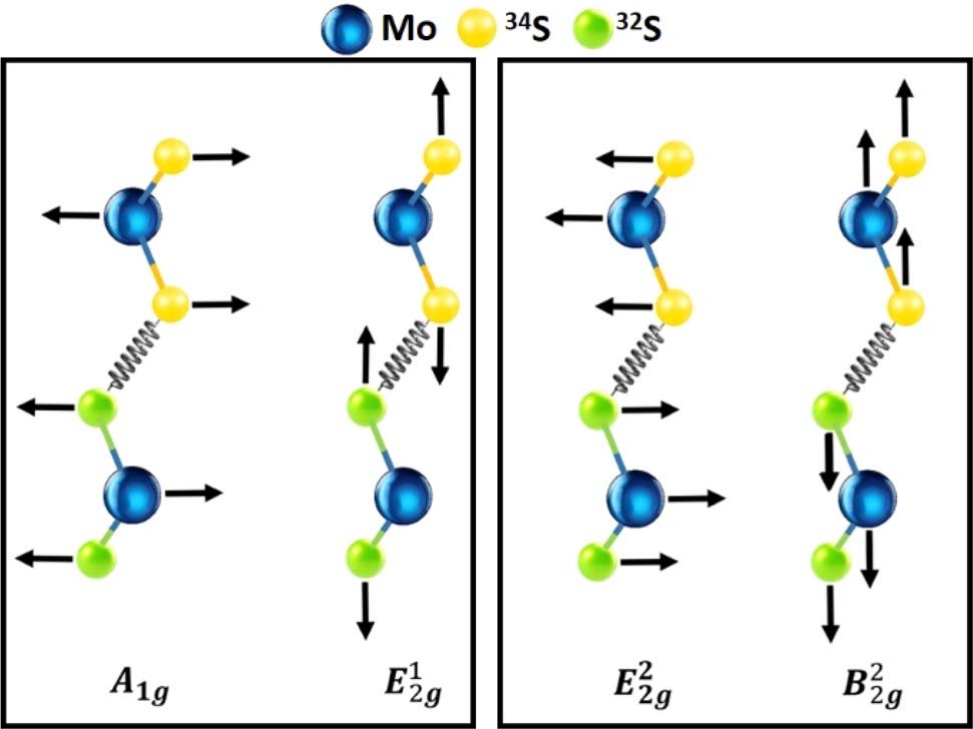
In-plane, out-of-plane (A_1g_ and E^1^_2g_) and inter-layer (E^2^_2g_ and B^2^_2g_) phonon modes of the IHS of MoS_2_(^32^S)/MoS_2_(^34^S).

The MoS_2_ MLs display a *P*6_3_/*mmc* space group symmetry and belong to a *D*_3h_ point group, revealing rotational and reflection symmetries.^35^ In the typical Raman spectra recorded in the out of the resonance condition of the incident laser radiation, the E^1^_2g_ (in-plane) mode and the A_1g_ (out-of-plane) mode occur typically at around 380 cm^−1^ at around 400 cm^−1^, respectively, as shown in the left panel of Fig. 2. Furthermore, low-frequency modes can be generally observed only in the multi-layered samples.^35,36^ These modes unveil the distinct vibrational signatures associated with the shear (S-E^2^_2g_) and breathing (B-B^2^_2g_) modes, offering insights into the interlayer interactions prevalent in layered materials. In case of double-layered IHS, the E^2^_2g_ mode, which indicates in-plane vibrational motions appears at 23 cm^−1^, while the B^2^_2g_ mode, which signifies the out-of-plane vibrations occurs at around 40 cm^−1^, (right panel Fig. 2).

From the relative intensity of the low frequency modes one can indicate the stacking order of the single layers.^37,38^ Fig. S2† displays the low-frequency shear and breathing Raman modes along with their fittings for a single isotope BL of the MoS_2_ and IHS in the AA stacking configuration (fitting parameters are presented in Table S1†). The analysis of the Raman maps of the grown samples revealed the prevalence of AA and AB stacking configurations (Fig. S4†). The AA stacking was indicated by the integral intensity ratio of the shear to breathing modes (B/S) to be about 3 ∼ 4.

As discussed above, the IHS is composed of the two overlapping MoS_2_ MLs containing different sulfur isotopes. The bottom layer, originating from ^32^S, displayed a triangular morphology, whereas the top layer grown using ^34^S, showed a pattern where randomly oriented crystals merged together. The resulting heterogeneous stacking pattern enveloped the entire triangular region, as illustrated in Fig. 1.

**Fig. 3:**
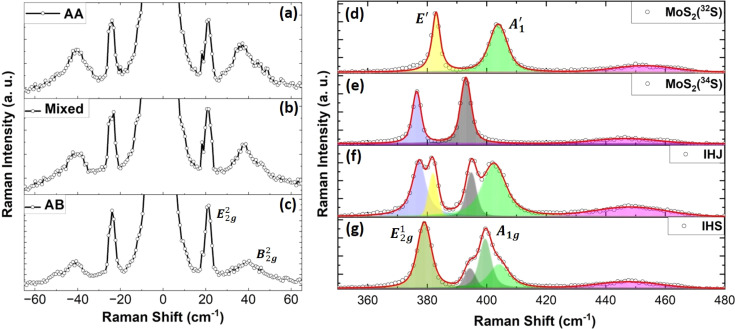
Low-frequency Stokes and anti-Stokes Raman spectra featuring the shear and breathing modes of IHS for (a) AA stacking (b) mixed phase and (c) AB stacking. (d) The actual stacking was estimated from the ratio of the integral intensities of the shear and breathing modes. Raman spectra along with respective deconvolution of the contributing modes: (e) MoS_2_(^32^S) ML, (f) MoS_2_(^34^S) ML, (g) IHJ, and (h) IHS.

The character of the stacking pattern was further investigated using low-frequency Raman measurements, which revealed spatial variation in the B/S ratio (Fig. 3(a–c)), indicative of the presence of mixed-phase stacking (∼2.5), *i.e.*, beyond AA and AB configurations with the different twisted angles. This observation highlights the heterogeneity in the stacking arrangements within the IHS, indicating variations in the interlayer coupling. This conclusion is further supported by analyzing the Raman spectral maps of the IHS, particularly the sum of S mode (which is significantly influenced by the stacking pattern). As shown in Fig. S3,† these maps confirm the presence of heterogeneous stacking within the IHS, indicating variations in the interlayer coupling.

As can be seen, the stacking order is a dominant effect which leads to the change of the intensity of the modes. In this case, the effect of the different isotopes is minor. One might expect that isotopes would affect the frequency of the B/S modes. However, this is not observed in the actual spectra, as the position remains essentially the same regardless of isotope content. This can be explained by considering that the B/S modes involve the mutual displacement of entire layers. Consequently, the relative change in mass due to different sulfur isotopes is minimal because molybdenum atoms are much heavier. Additionally, the B/S modes have low frequencies, so the relative change is negligible in absolute terms.

The typical Raman spectra in the low-frequency region of the pristine MLs of MoS_2_(^32^S) and MoS_2_(^34^S) are shown in Fig. 3(d) and (e), respectively. The spectra at the common fingerprint region are of the corresponding IHJ and IHS are displayed in Fig. 3(f) and (g), respectively. Each (d)–(f) graph also includes the respective deconvoluted peaks (using the PseudoVoigt functions, colored filled curves) and cumulative fits (red curves). The MoS_2_ MLs are characterized by distinct out-of-plane modes, 
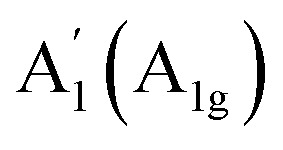
 and in-plane mode, E′ (E^1^_2g_). The Raman shift values of the 
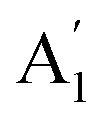
 and E′ for MoS_2_(^32^S) are found to be at around 405 cm^−1^ and 384 cm^−1^, respectively which is very close to the values reported for the MoS_2_ with natural sulfur, which contains around 95% of ^32^S and 4.25% of ^34^S. For MoS_2_(^34^S), the 
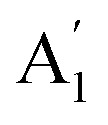
 and E′ modes occurred around 393 cm^−1^ and 377 cm^−1^. As expected, there is a significant red shift in both the Raman peaks in pristine MoS_2_(^34^S) compared to pristine MoS_2_(^32^S). This shift is due to the change in phonon frequencies caused by the heavier sulfur atoms in MoS_2_(^34^S). A detailed study of Raman analysis on pristine MoS_2_(^32^S) and MoS_2_(^34^S) has been reported in our previous work, where lattice dynamics are shown to be manipulated *via* sulfur isotope engineering.^26^

Raman shift of IHJ is characterized by a fingerprint of four peaks that can be distinctively de-convoluted into 
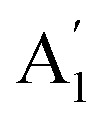
 and E′ modes of pristine MoS_2_(^32^S) and MoS_2_(^34^S) MLs. This pattern is further found to be converged in IHS where two distinct peaks of both A_1g_ and E^1^_2g_ can be seen around 400 cm^−1^ and 379 cm^−1^, respectively, roughly centralized between 
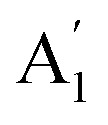
 and E′ modes of the pristine MLs of MoS_2_(^32^S) and MoS_2_(^34^S). Merging of 
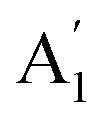
 and E′ modes of the two MoS_2_ MLs with the different isotopes of sulfur to form A_1g_ and E^1^_2g_ suggests a strong interlayer coupling between the layers.

Furthermore, the A_1g_ mode is found to be accompanied by two shoulder peaks on both sides corresponding to the position of the 
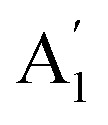
 mode of both pristine MLs. Interestingly, the complex Raman spectra of IHS resembles the behavior of isotopically engineered BL graphene, where the 2D mode was represented by three peaks: one corresponding to the position of 2D mode in ^12^C graphene ML, second corresponding to the position of 2D mode in ^13^C graphene ML and third roughly centralized between the position of 2D peak of ^13^C and ^12^C graphene MLs.^37^ This effect was understood by considering the possibility of the combination of the phonon modes in different layers of the BL.

Moreover, closer inspection of the spectra in the ^13^C/^12^C BL revealed that the intensities of the three peaks within the 2D band is in a ratio of 1 : 2 : 1. An analogous effect was observed in the IHS of the MoS_2_ for the A_1g_ mode but not for the E^1^_2g_ mode. The A_1g_ mode can be described by three peaks centered at 394, 399 and 404 cm^−1^. The intensity ratio of these peaks is approximately 1 : 2 : 1. This experimental observation thus suggest that the interlayer coupling is more important for the A_1g_ mode compared to the E^1^_2g_ mode.

The 2LA mode of MoS_2_ is relatively weak and quite broad which complicate the analysis. The Raman shift of the 2LA mode was found at 453 and 447 cm^−1^ for the MoS_2_(^32^S) and MoS_2_(^34^S), respectively. According to the expectation, the position of the Raman mode in IHS falls between that of MoS_2_(^32^S) and MoS_2_(^34^S) MLs. Nevertheless, the Raman shift of the 2LA mode in IHS is approximately 448 cm^−1^ which is slightly closer to the shift of the 2LA of the MoS_2_(^34^S) ML.

Note that the Raman modes in BL MoS_2_ are also found to be tuned *via* different parameters such as twist angle, polarization of light, *etc.*, but these effects are relatively small and ought to be suppressed in the IHS.^33,34,39,40^ The detailed fit of the Raman spectral maps together with the identification of the specific position on the sample is shown in Fig. S5.† The Raman shifts and full width at half maximum (FWHM) obtained from the fitting procedure are presented in Table S2.†

**Fig. 4:**
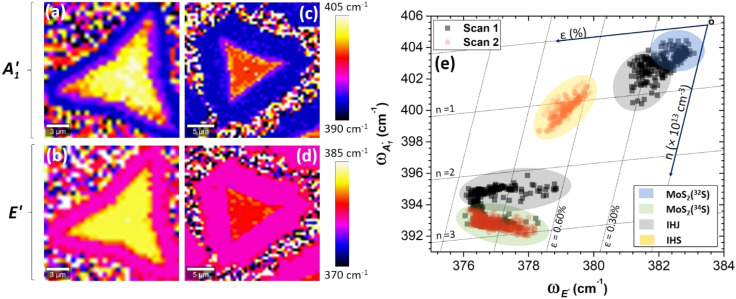
Raman spectral maps of the peak positions (Raman shifts) of partially and completely grown structures. (a) Out-of-plane mode 
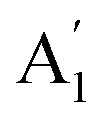
 and (b) in-plane mode E′ of the partially grown HS, (c) out-of-plane mode 
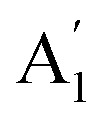
 and (d) in-plane mode E′ of the completely grown HS. Panel (e) presents correlation plots of the 
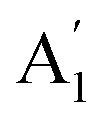
 and E′ modes for the different regions on the sample.

To analyze the distribution of Raman shift of 
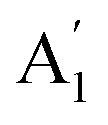
 and E′ modes across the IHJ and IHS, Raman spectral maps were collected across the sample. Fig. 4(a) and (b) show the Raman maps of the 
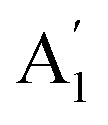
 and E′ for the partially grown IHJ, while Fig. 4(c) and (d) show the Raman maps for the completely grown IHS. To further understand the non-uniform distribution of the Raman shift between the edges and center of the sample, we performed a correlation analysis, which revealed the strain/doping present in the MoS_2_ layers.

The Raman correlation plots between frequencies of 
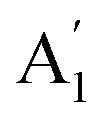
 and E′ Raman modes are shown in Fig. 4(e), where the labels “scan 1” (grey squares) and “scan 2” (pink circles) represent two different sets of the Raman mapping data recorded on purpose to distinguish the IHJ and fully grown IHS, respectively. All unique regions (MoS_2_(^32^S) and MoS_2_(^34^S) MLs, IHJ, and IHS) are presented by a specific color in the plot.

Usually, such correlation plots are used for the estimation of strain (*ε*) and doping concentration (*n*) in the 2D structures.^41,42^ The coordinates and scaling of the *ε*–*n* axis were adopted from previous reports, where the origin corresponds to the Raman modes of the pristine, mechanically exfoliated MoS_2_ ML with the natural isotope abundance of sulfur.^41^ The location of the clouds in isotopically modified structures is affected mainly by variation in the effective mass of the sulfur atoms, as can been seen from the clear separation of the clouds obtained for the isotopically pure MLs. Therefore, it is not trivial to directly evaluate the strain-doping experienced by the individual layers in the IHJ and IHS.

However, the shape and spread of the correlation clouds can still provide an interpretation of the relative distribution of strain and doping within the flake.^26^ For example, the cloud of MoS_2_(^32^S) in the blue region is evenly spaced in a circle while the cloud of MoS_2_(^34^S) in the green region is elongated through the strain axis. The character of the clouds suggests that while the strain and doping concentration in pristine MoS_2_(^32^S) ML is almost uniform across the flake, for pristine MoS_2_(^34^S) ML, the strain varies by about 0.5% across the analyzed region. Similarly, for the IHJ, the strain varies in the proximity of MoS_2_(^34^S) as evidenced by the lower elongated cloud of IHJ in the lower grey region. Interestingly, for IHS, it is found that both strain and doping concentration vary significantly over the mapped region by approximately 0.3% and 2 × 10^13^ cm^−3^, respectively.

In addition to the structural and lattice variations caused by the different masses of the sulfur isotopes in the HS, the optical responses of the isotopically modified HS were explored using PL mapping experiments. The PL spectra of MoS_2_ MLs in the visible region are usually composed of various contributions coming various types of neutral excitons and excitonic complexes. In general, the PL spectra typically consist of two main contribution from the A and B excitons, which are separated by the energy difference in the valence band originating from the spin–orbit coupling at the *K*-point in the Brillion zone.^26^ The B exciton is usually considered as a defect-induced peak which can be also enhanced through strain and doping, offering avenues for tailoring its optoelectronic properties.^43^

**Fig. 5:**
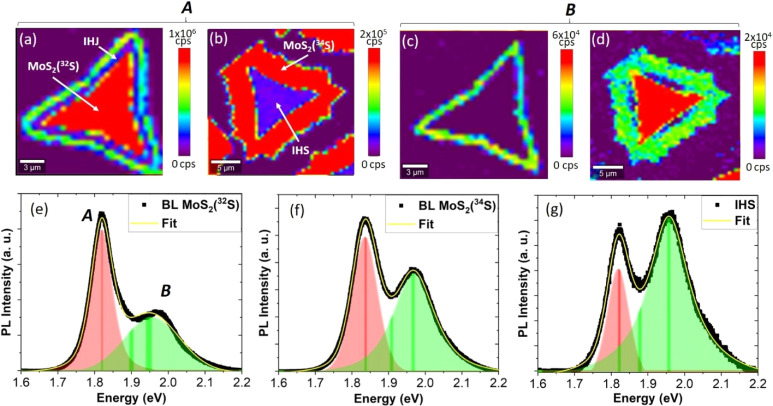
Maps of the PL intensity and the corresponding PL spectra at the specific regions of the sample. PL maps as the intensity (peak area) of the A exciton for (a) IHJ and (b) completely grown IHS. PL maps as the intensity (peak area) of B exciton for (c) IHJ and (d) completely grown IHS. PL spectrum along with the deconvoluted peaks of the A and B excitons for (e) BL MoS_2_(^32^S), (f) BL MoS_2_(^34^S), and (g) IHS.

Fig. 5(a) and (b) show the obtained PL map of the A exciton for IHJ and IHS, respectively. It can be seen that exciton A is pronounced in the outward and inward areas of the sample, which correspond to the pristine MoS_2_(^34^S) and MoS_2_(^32^S), respectively, while in the narrow IHJ region, the A exciton is diminished (Fig. 5(a)). A similar effect was found in the completely grown IHS, as shown in Fig. 5(b), where the outer region with the pristine MoS_2_(^34^S) exhibits pronounced A exciton as compared to the HS region in the middle of the crystal. This scenario is opposite in the case of B exciton, as can be seen in Fig. 5(c) and (d), respectively. This clearly shows that defect-related B exciton is dominant in IHJ and IHS compared to the isotopically pure pristine MLs.

Fig. S6† shows the PL spectra of isotope-modified pristine MoS_2_ MLs along with their deconvolution into peaks attributed to the A and B excitons (a detailed study on exciton dynamics of isotope-modified MoS_2_ MLs is presented in our previous report.^26^) In the present work, a comparative analysis of the PL spectra is discussed for BL MoS_2_(^32^S) and BL MoS_2_(^34^S) along with the IHS. Fig. 5(e–g) shows the PL spectra from the pristine BL MoS_2_(^32^S), BL MoS_2_(^34^S), and IHS, respectively. The final fit is shown as the yellow line (the fitting parameters are presented in Table S3†), while the contributing peaks are presented as solid color curves.

As expected, the energy of A and B exciton are slightly smaller for BL MoS_2_(^32^S) than for BL MoS_2_(^34^S) given the changes of the electronic structure induced by the sulfur isotope. However, it is interesting that the A and B exciton positions in IHS are matching those in the case of BL MoS_2_(^32^S). This finding is important as it implies that in IHS, the excitons are transferred from MoS_2_(^34^S) to MoS_2_(^32^S) and consequently the emission is observed only from the MoS_2_(^32^S) layer.

The A exciton is strongly quenched in pristine BLs as well as in IHS. Such exciton quenching can be attributed to the significant interlayer coupling in BL MoS_2_ as the interaction between adjacent layers alters the electronic band structure and affects the efficiency of exciton formation, reducing the population of the A excitons. Consequently, the interplay of interlayer coupling and charge transfer phenomena diminishes the occurrence of A excitons in BL MoS_2_.^13,44^

The intensity ratio of the A exciton in BL compared to ML with the same isotope composition is estimated to be between 0.04 and 0.1, suggesting stronger interlayer coupling in BL MoS_2_(^34^S) as compared to BL MoS_2_(^32^S). However, for IHS, these ratios are estimated to be around 0.016 and 0.03, as compared to ML MoS_2_(^34^S) and ML MoS_2_(^32^S), respectively. This indicates even stronger interlayer coupling in IHS yielding strong A exciton quenching. The positions of the A and B excitons are found to be almost similar for the BL MoS_2_(^32^S) and IHS while blue-shifted for BL MoS_2_(^34^S), which can be attributed to bandgap changes and surface effects resulting from the presence of heavier isotopes. This observation also suggests that in case of IHS, the energy of excitons is transferred between adjacent ML, *i.e.*, from MoS_2_(^34^S) to MoS_2_(^32^S). It can also be seen that the ratio of the exciton integral intensity, B/A varies significantly between BLs and IHS, *i.e.*, BL MoS_2_(^32^S) ∼0.9, BL MoS_2_(^34^S) ∼1.5, and IHS ∼3.6. In the IHS, the slightly larger bandgap observed in MoS_2_(^34^S) MLs can result in a higher energy threshold for exciton formation, favoring the formation of B excitons over A excitons. Additionally, stacking layers with different isotopes can induce strain and lattice mismatch effects in adjacent MLs. These effects may distort the crystal lattice and modify the electronic band structure, impacting exciton formation. This aligns with recent reports that clearly demonstrate defect levels can be tuned in HS.^45^

A detailed mapping of the A and B excitons within an appropriate narrow range scale revealed variations in their intensities as shown in Fig. S7.† Specifically, regions where the B exciton exhibited enhanced intensity corresponded to the areas where the A exciton intensity was relatively diminished. These variations in the A and B exciton intensity closely correlate with regions where Raman mapping indicated the variations in the shear mode intensity (see Fig. S4(a–d)).† This correlation suggests that the stacking heterogeneity observed in the Raman mapping is also manifested in the A and B exciton intensity ratios. Explicitly, regions with AB stacking exhibit a slightly increased B exciton intensity, consistent with previous reports.^46,47^ This is further corroborated in the single-point PL spectra taken from zones corresponding to the different stacking (Fig. S7(e)).† Even though the PL shows variations in the A/B exciton intensity ratio, the variation is relatively small with respect to the changes caused by different sulfur isotopes discussed above.

**Fig. 6:**
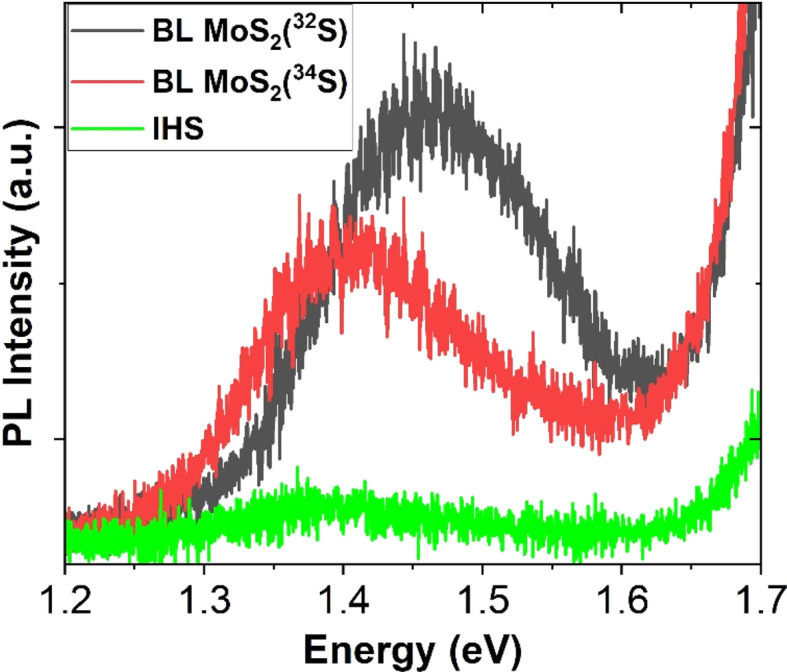
PL spectra in the I band region for the BL MoS_2_(^32^S), BL MoS_2_(^34^S), and IHS.

Another important fingerprint in the PL spectra of the TMDC BLs is the I band, which appears as a result of the momentum-assisted transition across the indirect bandgap between the *Γ* and *K* points in the Brillion zone.^48,49^Fig. 6 shows the comparison of the I band emission for the pristine BLs and IHS.

For the MoS_2_(^34^S) and MoS_2_(^32^S) BLs, the intensities of the I band is comparable and significant, while the I band completely vanishes in the case of IHS. This is an important finding as it points to a suppression of the intraband process due to the mixed isotopic occurrence of the IHS.

Notably, we demonstrated by Raman spectroscopy that the coupling between the layers in the IHS is strong and thus the vanishing of the I band cannot be attributed to a weak coupling of the layers. Thus, the miniscule signature of the I in the IHS is consistent with our conclusions that due to the specific electronic structures of the isotopically engineered layers, the excitons in IHS are preferentially transferred from MoS_2_(^34^S) to MoS_2_(^32^S) layer. Consequently, other processes, including formation of interlayer excitons in IHS are strongly suppressed.

We note that isotopic effects in adjacent layers of IHS can also lead to non-uniform strain distributions as indicated by the Raman correlation plot, which may also hinder the efficient generation or detection of interlayer excitonic states contributing to the I band. Therefore, despite strong interlayer coupling, the I band could cease in IHS.

To elucidate further on interlayer interactions, TRPL measurements were employed to explore the exciton dynamics in the isotope-modified pristine MLs and IHS.

**Fig. 7:**
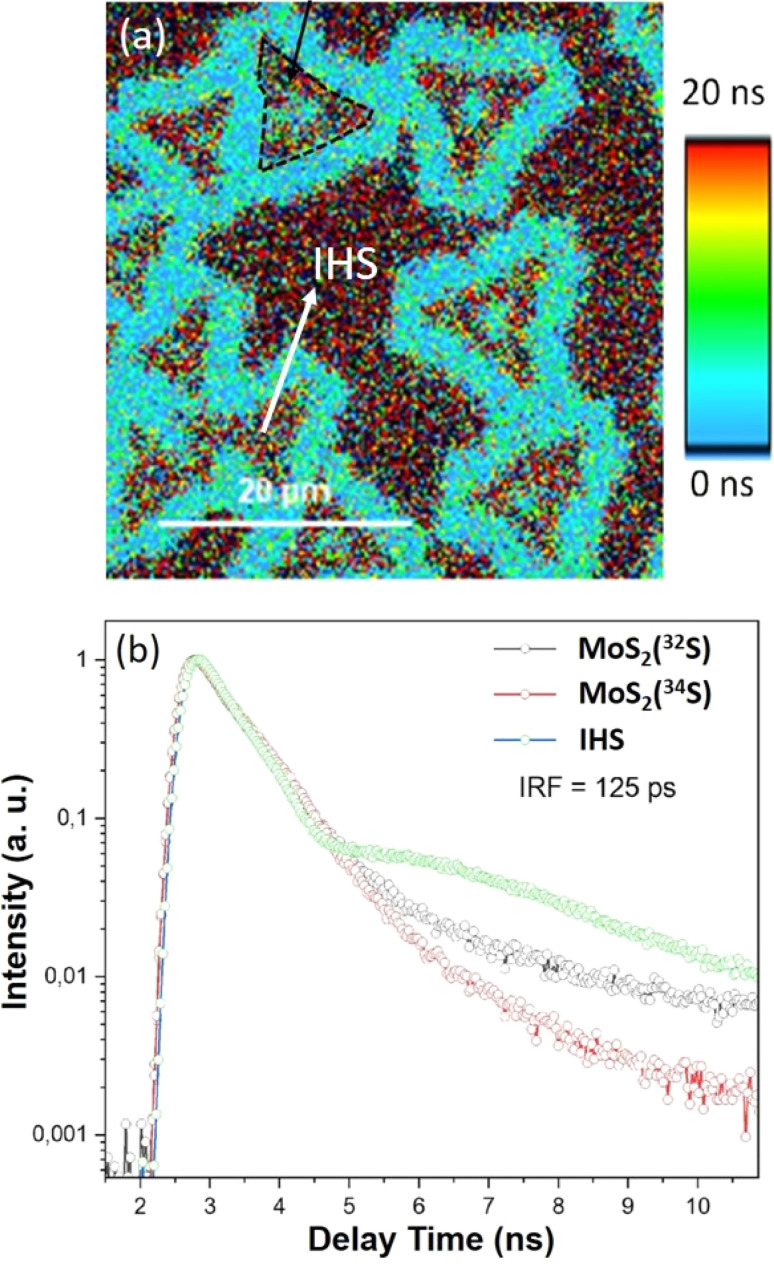
(a) TRPL mapping of the as-grown IHS and (b) the corresponding transient decay profiles at the region of interest, as identified in the (a).

The obtained TRPL micrograph is shown in Fig. 7(a), and the corresponding deconvoluted decay profiles at the regions of interest on MoS_2_(^32^S) and MoS_2_(^34^S) MLs, and the IHS are shown in Fig. 7(b). Usually, the lifetimes of the MoS_2_ MLs are in the order of 10^−1^ ns; the MoS_2_(^32^S) and MoS_2_(^34^S) MLs exhibit a dominant decay constant (*τ*_1_) of ∼0.69 ± 0.1 and 0.70 ± 0.1 ns, which is consistent with the previous reports.^26,50,51^ The decay profile of the IHS exhibits a slightly faster decay channel of a lifetime, *τ*_1_ ∼ 0.60 ± 0.1 ns with a strong superimposition of a slower decay channel of a lifetime, *τ*_2_ ∼ 5.0 ± 0.1 ns. The faster *τ*_1_ and appearance of *τ*_2_ are due to isotopically labeled homojunction formation, where *τ*_1_ represents the intra-layer carrier recombination and *τ*_2_ reflects the recombination of the charges after intersystem crossing. A slower decay rate has been reported in mechanically exfoliated BLs of MoS_2_ as compared to ML, where *τ*_2_ is more dominant.^52^ The contrary faster decay in the IHS can be understood by considering the energy transfer between the layers induced by a slight difference in the bandgap of the MoS_2_(^32^S) and MoS_2_(^34^S).

## Conclusions

Using the two-step CVD growth process, we successfully prepared isotopically engineered heterostructures and heterointerfaces with controlled sulfur isotope content. Optical images and Raman spectra analysis of the shear and breathing modes indicated variations in the layer stacking order, with predominantly AA and AB configurations. The frequencies of the 
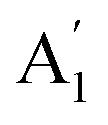
 and E′ modes were notably influenced by the sulfur isotope content and the configuration of the HS. In the IHJ, the principal Raman active modes appeared in the spectra as for two independent MLs with a certain shift due to the different sulfur isotope mass, while the spectra of the IHS revealed only two main modes (A_1g_ and E^1^_2g_). The A_1g_ mode was found to be composed of three overlapping bands, highlighting strong interlayer phonon coupling between adjacent layers in the IHS. The Raman spectra of IHS and isotopically engineered BL graphene exhibit similar behavior, with the 2D mode showing three peaks, corresponding to different phonon modes in the layers. Furthermore, the intensity ratio of 1 : 2 : 1 observed for the A_1g_ mode suggests stronger interlayer coupling for this mode compared to the E^1^_2g_ mode. The PL intensity analysis of the MoS_2_(^34^S) and MoS_2_(^32^S) BLs revealed significant quenching of the A excitons compared to BLs composed of isotopically identical layers, with a relatively enhanced B exciton intensity. The positions of PL bands correlated closely with sulfur isotope content, facilitating analysis of IHS PL dominated by emission from the MoS_2_(^32^S) layer due to its smaller bandgap.

Moreover, a notable suppression of the I band in IHS suggested limited intralayer exciton formation. Despite some observed variations in stacking order, the robustness of the isotope effects supports our conclusions. Finally, time-resolved photoluminescence (TRPL) measurements demonstrated faster lifetimes in IHS, indicating a nuanced interplay of interlayer coupling dynamics in the isotopically modified BLs.

These findings highlight the great potential of isotope engineering in TMDC-based HS for advancing new concepts in optoelectronics and quantum information technologies, particularly in tailoring and enhancing emission mechanisms. In a broader context, this approach allows for precise tailoring of the electronic structure of materials with strongly confined quasiparticles at the nuclear level, rather than altering electronic states through chemical modifications.

## Data availability

Data for this article are available at Zenodo (https://zenodo.org/) at https://zenodo.org/records/13857956.

## Conflicts of interest

The authors declare no conflict of interest.

## Supplementary Material

NA-OLF-D4NA00897A-s001
