# Supplementary material for: Sulfur isotope engineering in heterostructures of transition metal dichalcogenides
Source: Nanoscale Adv. 2025 Jan 13;7(5):1276–86. doi: 10.1039/d4na00897a (PMC11748049; doi:10.1039/d4na00897a)
Supplement: NA-007-D4NA00897A-s001 [file NA-007-D4NA00897A-s001.pdf]

Supplementary Information (SI) for Nanoscale Advances.
This journal is © The Royal Society of Chemistry 2025

Sulfur Isotope Engineering in Heterostructures of Transition Metal Dichalcogenides

Vaibhav Varade†, Golam Haider‡, Martin Kalbac‡*, Jana Vejpravova†*

†*Department of Condensed Matter Physics, Faculty of Mathematics and Physics, Charles University, Ke Karlovu 5, 121 16, Prague 2, Czech Republic*

‡*Department of Low-Dimensional Systems, J. Heyrovsky Institute of Physical Chemistry, Dolejskova 3, 182 23, Prague 8, Czech Republic*

E-mail: martin.kalbac@jh-inst.cas.cz

E-mail:jana.vejpravova@matfyz.cuni.cz

Supplementary Information

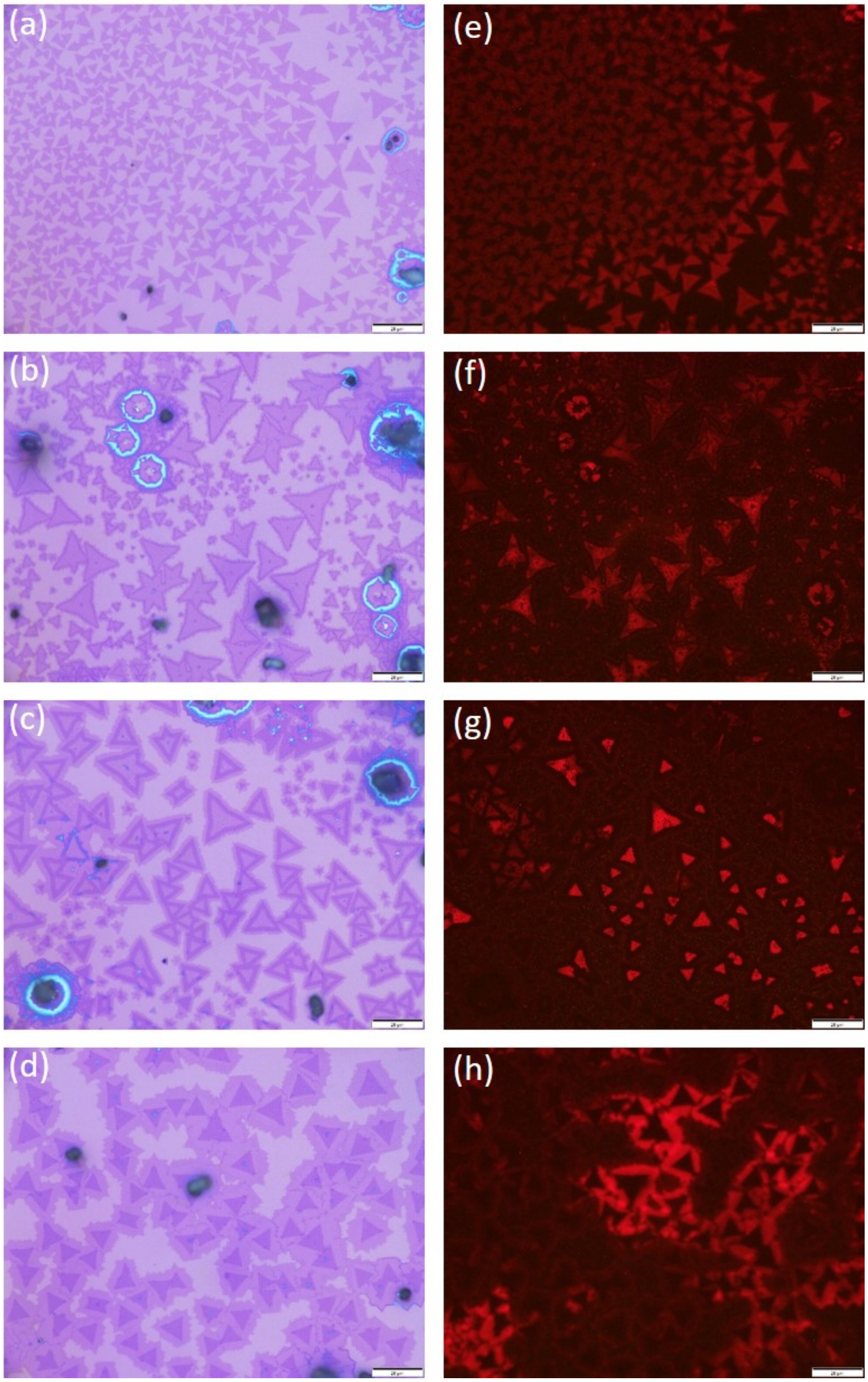

Figure S1: (a-d) Microscopic optical images depicting the growth pattern of MoS_2(^{34}S/MoS_2(^{32}S) IHS and their (e-h) corresponding PL images under green light.

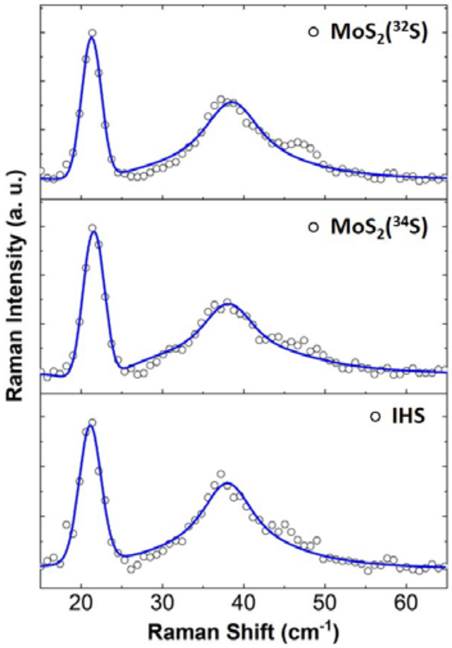

Figure S2: Raman spectra depicting shear and breathing modes, E_{2g}^{2} and B_{2g}^{2} along with fitting for BL MoS_2(^{32}S), BL MoS_2(^{34}S) and IHS.

Sample	Raman Modes	Raman shift (cm^{-1})	FWHM (cm^{-1})	Integral Intensity ($cps.cm^{-1}$)
BL MoS_2(^{32}S)	E_{2g}^{2}	21	3	4201
	B_{2g}^{2}	39	10	15019
BL MoS_2(^{34}S)	E_{2g}^{2}	22	3	2410
	B_{2g}^{2}	38	10	10025
IHS	E_{2g}^{2}	21	3	1037
	B_{2g}^{2}	38	9	3086

Table S1: Fit parameters of E_{2g}^{2} and B_{2g}^{2} from the fitting of **Figure S3.**

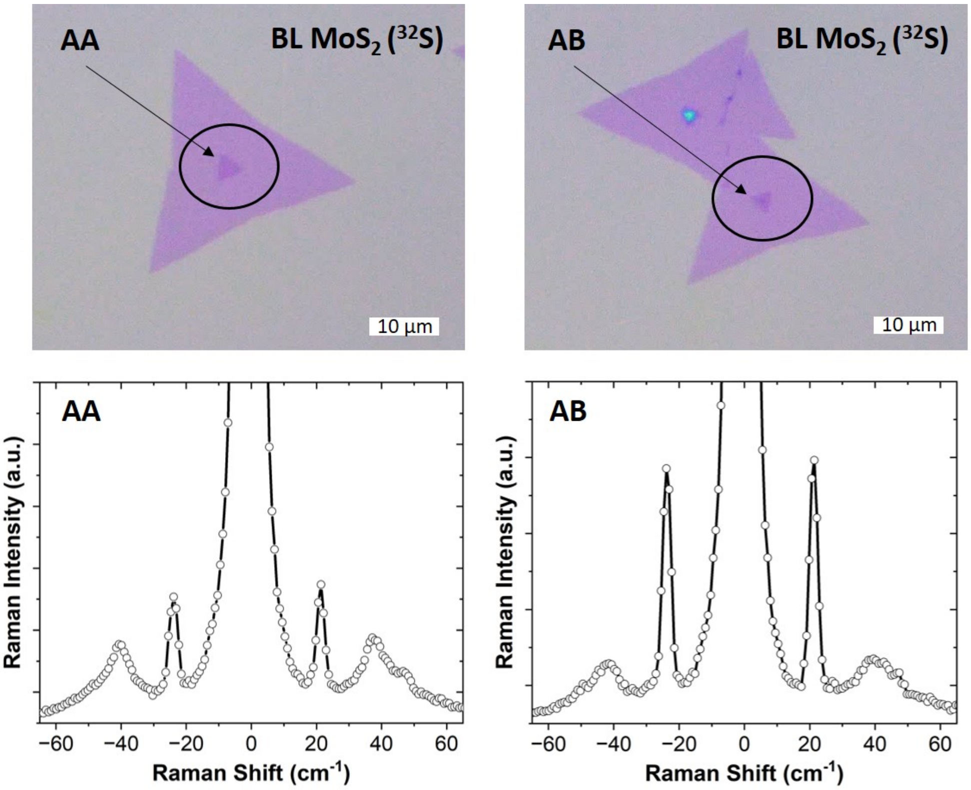

Figure S3: Optical images of BL MoS_2(^{32}S) exhibiting AA and AB stackings along with their corresponding low-frequency Raman modes.

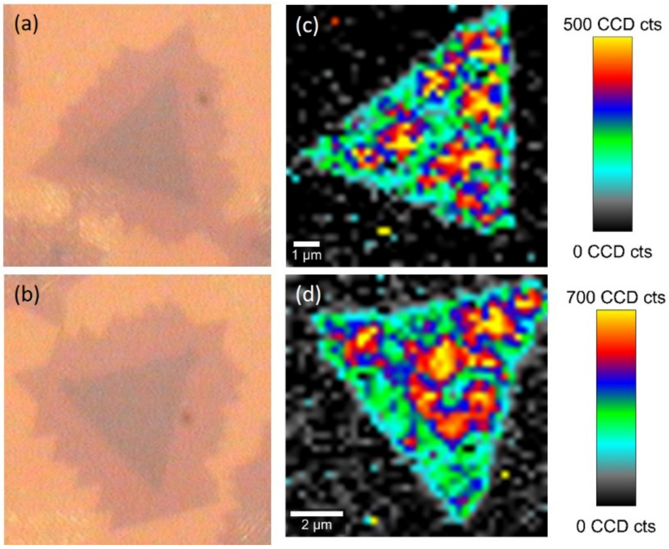

Figure S4: (a) and (b) shows the optical images of two IHS along with their Raman mapping across the sum of shear (S) mode in (c) and (d), respectively.

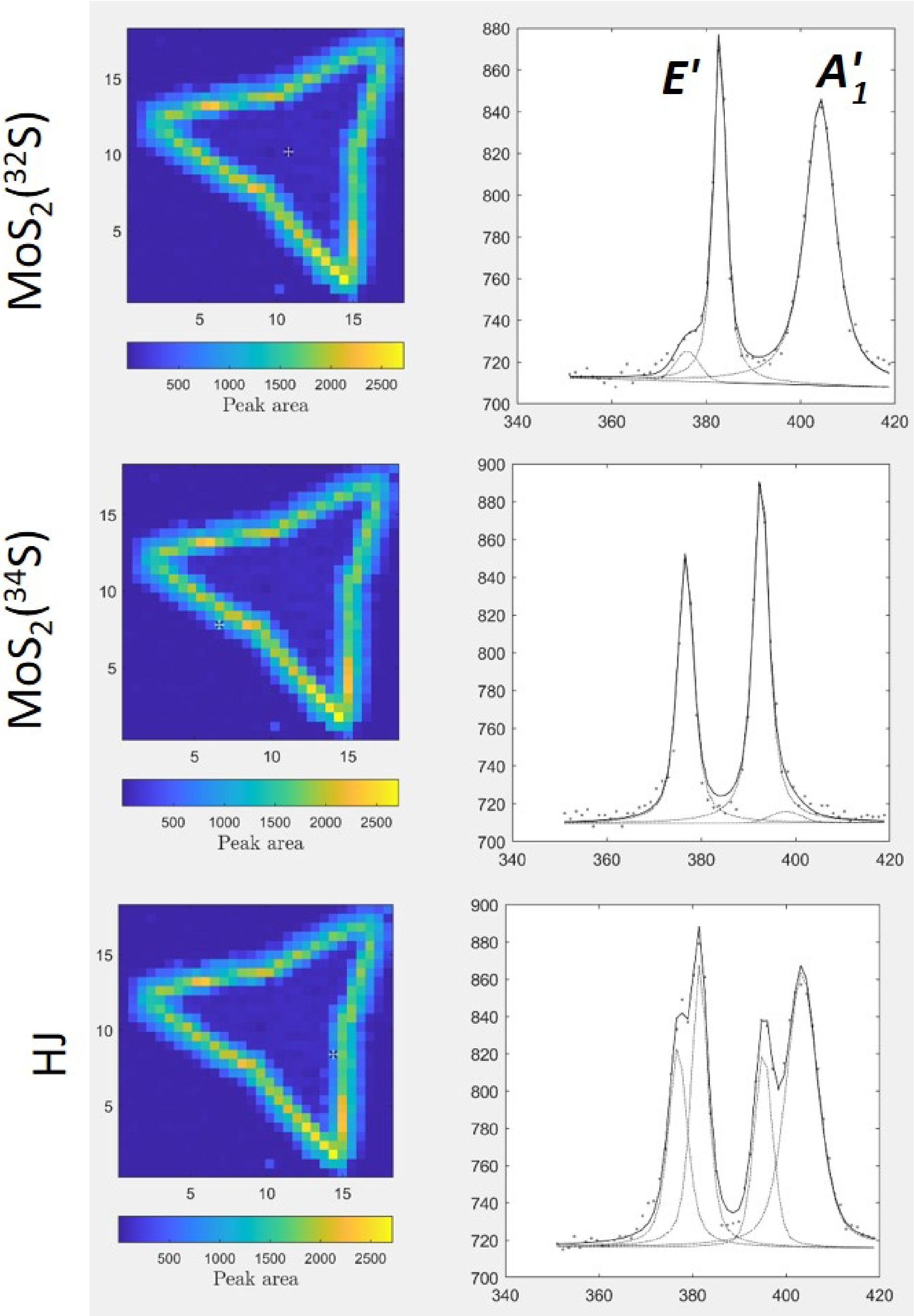

Figure S5: Spatial map of $MoS_2(^{32}S)$ and $MoS_2(^{34}S)$ depicting IHJ along with two peak fittings of the Raman spectra from the position depicted in the cursor.

Sample	Raman Modes	Raman shift (cm^{-1})	FWHM (cm^{-1})
	E'	383	3
MoS_2(^{32}S)	A_1'	404	7
	$2LA$	453	24
	E'	377	4
MoS_2(^{34}S)	A_1'	393	4
	$2LA$	447	25
	E'	377	6
	E'	382	4
IHJ	A_1'	395	4
	A_1'	402	10
	$2LA$	449	28
	E_{2g}^1	379	6
	A_{1g}	394	4
IHS	A_{1g}	400	5
	A_{1g}	404	8
	$2LA$	448	25

Table S2: Fitting parameters for the Raman spectra of ML MoS_2(^{32}S), ML MoS_2(^{34}S), IHJ, and IHS are shown in **Figure 3(d-g)**.

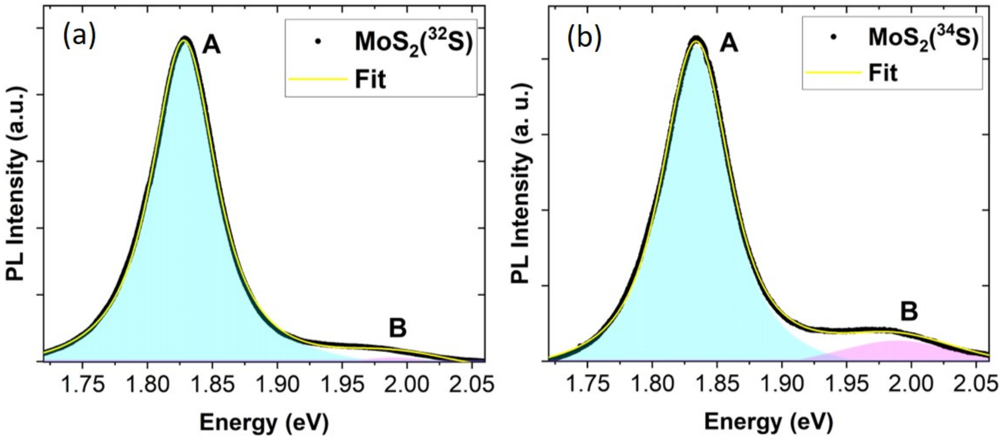

Figure S6: PL spectra along with fitting in yellow line and deconvolution of *A* and *B* excitons for (a) ML MoS_2(^{32}S) and (b) ML MoS_2(^{34}S).

Sample	Exciton	Exciton Energy (eV)	FWHM (meV)	Integral Intensity (cps.eV)
BL $MoS_2(^{32}S)$	*A*	1.82	71	381
	B	1.96	181	336
BL $MoS_2(^{34}S)$	*A*	1.84	83	519
	B	1.97	138	760
IHS	*A*	1.82	72	40
	B	1.96	140	147

Table S3: Fitting parameters for the PL spectra of BL $MoS_2(^{32}S)$, BL $MoS_2(^{34}S)$, and IHS in **Figure 5**.

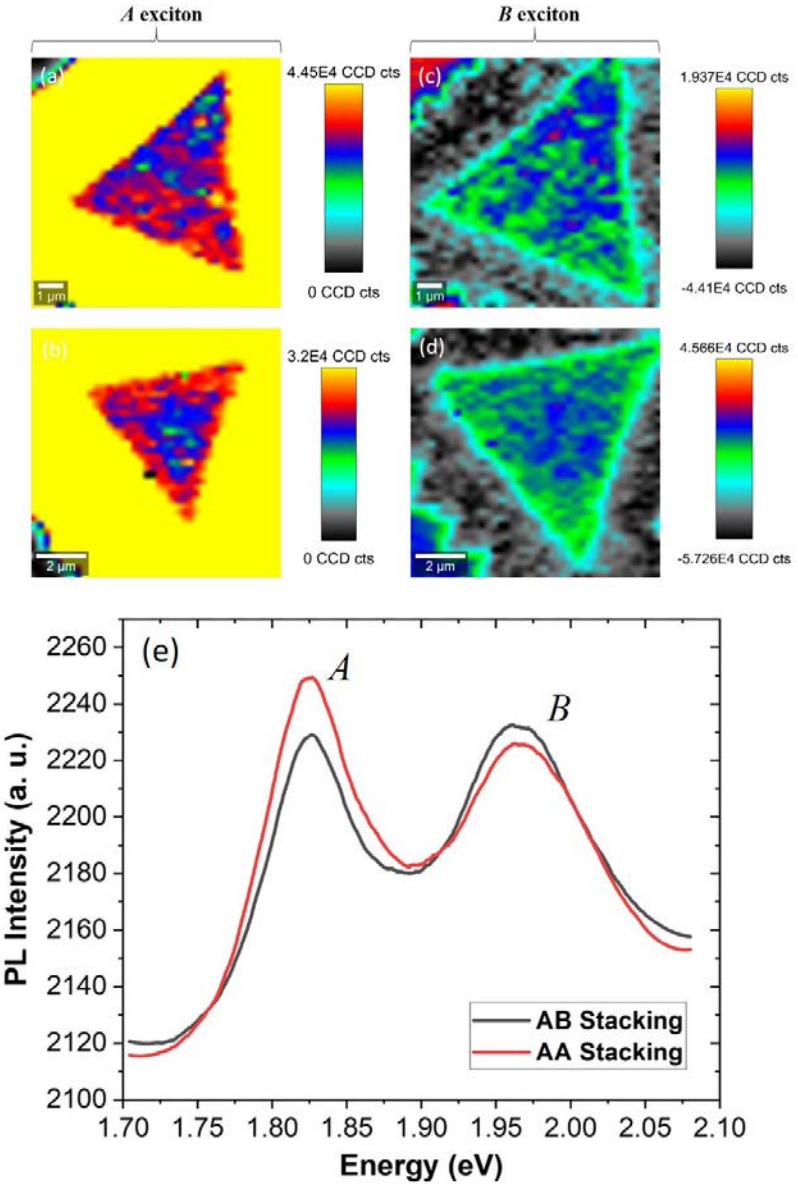

Figure S7: Narrow range scaled PL Mapping of (a-b) A exciton and (c-d) B exciton in two different IHS correlating the heterogeneous stacking indicated by Raman mapping of shear mode in **Figure S4**. (e) Single PL spectra taken from AB and AA stacking zone of (b).
